# Effects of Thermal Exposure on the Microstructure and Mechanical Properties of a Ti–48Al–3Nb–1.5Ta Alloy via Powder Hot Isostatic Pressing

**DOI:** 10.3390/ma17040794

**Published:** 2024-02-07

**Authors:** Zhenbo Zuo, Rui Hu, Qingxiang Wang, Zitong Gao, Xian Luo, Yunjin Lai, Sa Xue, Min Xiang, Xiaohao Zhao, Shaoqiang Li

**Affiliations:** 1 State Key Laboratory of Solidification Processing, Northwestern Polytechnical University, Xi’an 710072, China; zuozhenbo@mail.nwpu.edu.cn (Z.Z.);; 2 Sino-Euro Materials Technologies of Xi’an Co., Ltd., Xi’an 710018, China

**Keywords:** Ti–48Al–3Nb–1.5Ta, thermal exposure, oxidation, stability

## Abstract

Research on how thermal exposure affects the microstructure and mechanical properties of the Ti–48Al–3Nb–1.5Ta (at. %) alloy, which is prepared via powder hot isostatic pressing (P–HIP), is essential since this low-density alloy shows promise for use in high-temperature applications, particularly for aero-engines, which require long-term stable service. In this study, a P–HIP Ti–48Al–3Nb–1.5Ta (at. %) alloy was exposed to high temperatures for long durations. The phase, microstructure and mechanical properties of the P–HIP Ti–48Al–3Nb–1.5Ta alloy after thermal exposure under different conditions were analyzed using XRD, SEM, EBSD, EPMA, TEM, nanomechanical testing and tensile testing. The surface scale is composed of oxides and nitrides, primarily Al_2_O_3_, TiO_2_, and TiN, among which Al_2_O_3_ is preferentially generated and then covered by rapidly growing TiO_2_ as the thermal exposure duration increases. The nitrides appear later than the oxides and exist between the oxides and the substrate. With increasing exposure temperature and duration, the surface scale becomes more continuous, TiO_2_ particles grow larger, and the oxide layer thickens or even falls off. The addition of Ta and Nb can improve the oxidation resistance because Ta^5+^ and Nb^5+^ replace Ti^4+^ in the rutile lattice and weaken O diffusion. Compared with the P–HIP Ti–48Al–3Nb–1.5Ta alloy, after thermal exposure, the grain size does not increase significantly, and the γ phase increases slightly (by less than 3%) with the decomposition of the α_2_ phase. With increasing thermal exposure duration, the γ phase exhibits discontinuous coarsening (DC). Compared with the P–HIP Ti–48Al–3Nb–1.5Ta alloy, the hardness increases by about 2 GPa, the tensile strength increases by more than 50 MPa, and the fracture strain decreases by about 0.1% after thermal exposure. When the depth extends from the edge of the thermally exposed specimens, the hardness decreases overall.

## 1. Introduction

The TiAl alloy is a promising high-temperature material for aerospace applications because of its low density, exceptional creep and high-temperature oxidation resistance [1,2,3,4,5,6]. A stable structure and mechanical performance at high temperatures for a long service life are essential for TiAl alloys to be used as high-temperature structural materials in equipment that requires high safety and stability [7]. It has been demonstrated that under prolonged thermal exposure at high temperatures, the microstructure and phase composition of the TiAl alloy undergo changes, including continuous coarsening, discontinuous coarsening (DC) of the lamella, and the production of equiaxed grains [8]. The related phase transformations are α_2_ lamella decomposition (vertical and parallel decomposition) [9,10] and B2 phase transformation (B2 → ω and B2 → γ) [11]. The microstructural evolution of the TiAl alloy at high temperatures is affected by the composition, structure, temperature, duration and other factors, and long-term thermal exposure promotes microstructural evolution [12]. Essentially, thermodynamics determine whether the microstructure is stable at high temperatures, whereas dynamics control the rate and degree of evolution [3]. The evolution of the microstructure directly affects the mechanical properties of TiAl alloys. For instance, the hardness of 47Al–2Nb–2Mn–0.8%TiB_2_ (volume fraction) and Ti–45Al alloys decreases when the lamella spacing coarsens [13].

The oxidation resistance of the TiAl alloy is intimately tied to the Al_2_O_3_ protective layer, which cannot fully develop although the TiAl alloy has a high Al content. Meanwhile, TiO_2_ grows rapidly but cannot offer long-term antioxidative protection [14]. Therefore, the oxidation resistance of traditional TiAl alloys is insufficient above 800 °C [15,16]. 

Previous research [17,18,19,20,21,22,23,24,25,26,27] reveals that the addition of alloying elements, such as W, Mo, Ta and Nb, can prevent the lamella from coarsening and significantly improve the high-temperature oxidation resistance of the TiAl alloy; among these, the effects of Ta and Nb are particularly notable. Nb can efficiently improve the outward diffusion rate of Al while reducing the growth rate of TiO_2_ particles and the diffusion rate of O [24,28,29]. With increasing Nb concentration, the TiAl alloy’s high-temperature oxidation resistance increases linearly [30]. The addition of Ta limits the solubility of O in the TiAl alloy and prevents TiO_2_ from growing at high temperatures because of its low diffusion coefficient [31].

According to previous studies [32,33,34], the addition of Ta to TiAl alloys can improve their mechanical properties, including their tensile strength, compressive strength and hardness, due to solution strengthening and refinement of the massive γ phase by controlling metastable microstructural evolution [35,36,37]. Ta and Nb elements can extend the cooling rate requirement for the α → γ_m_ massive transformation to avoid the quenching of cracks caused by an excessive cooling rate [38]. The solution effect of Ta addition is stronger than that of Nb, which can effectively increase creep resistance without changing the phase transformation path [39], and Ta can improve the plasticity of the TiAl alloy more obviously than Nb [40]. In addition, Ta and Nb, which promote massive transformation and inhibit lamella transformation at low temperatures, have low diffusion coefficients in the α and γ phases. Segregation occurs with the addition of Ta and Nb elements [41,42], although they improve the high-temperature performance and oxidation resistance of the TiAl alloy. However, powder metallurgy is an ideal preparation method because it achieves chemical homogeneity and fine grains while avoiding segregation [43]. 

The present work is focused on the investigation of the thermal exposure characteristics of a new powder metallurgical TiAl alloy containing Nb and Ta prepared by powder hot isostatic pressing (P–HIP). Ti–48Al–3Nb–1.5Ta (at. %) alloy, as a hyperperitectic TiAl with almost no B2 phase, can prevent microstructure instability and performance reduction from B2 decomposition at high temperatures. Proper addition of Ta and Nb can improve the oxidation resistance and mechanical performance at high temperatures. The improved segregation of Nb and Ta and the promotion of microstructure homogeneity through the P–HIP process are expected to further elevate the service temperature of TiAl alloys. The application of the material at high temperatures is directly impacted by alterations in the microstructure and mechanical properties with long-term thermal exposure. However, the thermal exposure characteristics of P–HIP Ti–48Al–3Nb–1.5Ta alloy are currently unclear. For application guidance, such as for aero-engines used in harsh environments requiring long-term stable service, it is vital to study the effects of thermal exposure on the microstructure and mechanical properties of the P–HIP Ti–48Al–3Nb–1.5Ta alloy. In this study, the surface morphology, microstructural evolution and mechanical performance of the P–HIP Ti–48Al–3Nb–1.5Ta (at. %) alloy during thermal exposure were investigated and clarified.

## 2. Materials and Methods

**Figure 1:**
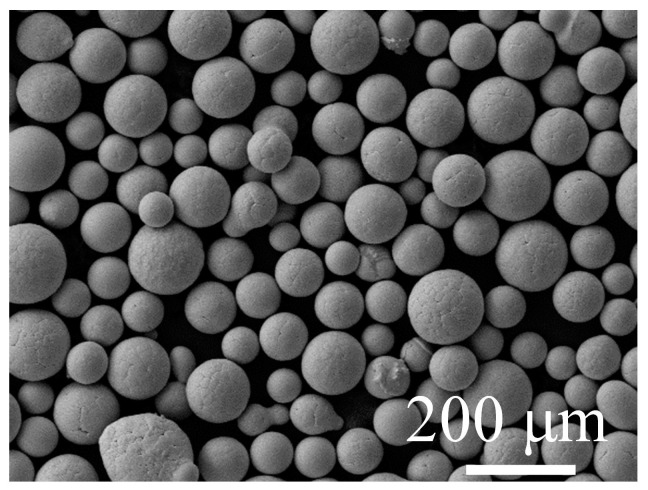
The typical morphology of SS-PREP^®^ Ti−48Al−3Nb−1.5Ta powders in the range of 45–250 μm.

Ti–48Al–3Nb–1.5Ta (at. %) prealloyed powders were prepared using SS-PREP^®^ from Sino-Euro Materials Technologies of Xi’an Co., Ltd. (Sino-Euro, Xi’an, China) and then sieved into 45–250 μm sized powders. The powders were mostly perfect spherical in shape, as shown in Figure 1. The specific prealloyed powders were poured into steel capsules and degassed at 400 °C for 4 h to remove air. The stem at the top of the capsule was welded when the vacuum of the capsule reached $1\times 10^{-4}$ Pa. The sealed capsules were placed in the HIP furnace and heated to 1200 °C with a heating rate of 4 °C/min and pressurized up to 150 MPa for 4 h, followed by cooling at 5 °C/min to room temperature.

The 10 × 10 × 10 mm specimens were cut from the P–HIP Ti–48Al–3Nb–1.5Ta alloy billet after the capsules were removed. Some specimens were put into quartz tubes and sealed before exposure (sealed specimens), while the others were exposed directly (unsealed specimens). The unsealed specimens were used to analyze the surface morphology and scale constitution, and the sealed specimens were used to analyze the microstructural evolution and mechanical properties. The sealed specimens with the size of φ8 × 67 mm after thermal exposure were machined into specimens with a diameter of 3 mm and a gauge length of 15 mm for tension testing at room temperature. The exposure temperatures were 800 °C, 850 °C and 900 °C and the duration was 100 h, 300 h, 500 h, 800 h or 1000 h.

The phase constituents were analyzed using X-ray diffraction (XRD, Bruker D8 DISCOVER A25, Bruker, Billerica, MA, USA) with Co Kα radiation at a rate of 2.5°/min from 20° to 90° at room temperature. The surface morphology, microstructure and element distribution were analyzed using scanning electron microscopy (SEM, ZEISS Sigma 300, Oberkochen, Germany) equipped with an energy-dispersive X-ray spectrometer (EDX, ZEISS Sigma 300, Oberkochen, Germany), an electron probe microanalyzer (EPMA, SHIMADZU EPMA-1720, Kyoto, Japan) and a transmission electron microscope (TEM, FEI Talos F200X TEM, Hillsboro, OR, USA). The crystallographic features were further analyzed using electron backscattered diffraction (EBSD, ZEISS Sigma 300, Oberkochen, Germany). The hardness was analyzed using a nanomechanical testing system (Hysitron TI-950, Bruker, Billerica, MA, USA) with a 10 mN force. The tensile performance was determined using a microcomputer-controlled electron universal testing machine (CMT5105, Sansi Yongheng, Ningbo, China) according to GB/T 228.1-2021 (Chinese Standard) [44].

The 10 × 10 × 10 mm sealed specimens were cut in half and mechanically mounted in epoxy resin, polished with SiC abrasive papers and a polishing cloth and, finally, polished by a vibratory polisher; these were then used for SEM and EBSD analyses on internal cross-sections. The specimens for TEM were first cut into a 400 μm thick slice from the middle of the 10 × 10 × 10 mm sample, then manually ground to a 50 μm thickness with SiC abrasive papers and, finally, subjected to ion milling. The hardness inside the specimens was obtained as arithmetic mean values from ten testpoint measurements approximately evenly distributed along a straight line of one 10 × 10 × 10 mm sample with the same preparation method as for EBSD. Near-surface hardness testing was conducted on unsealed specimens along the cross-section from the edge with an interval of 3.5 μm for a total 10 testpoints.

## 3. Results and Discussions

### 3.1. Microstructures of the P–HIP Ti–48Al–3Nb–1.5Ta Alloy

**Figure 2:**
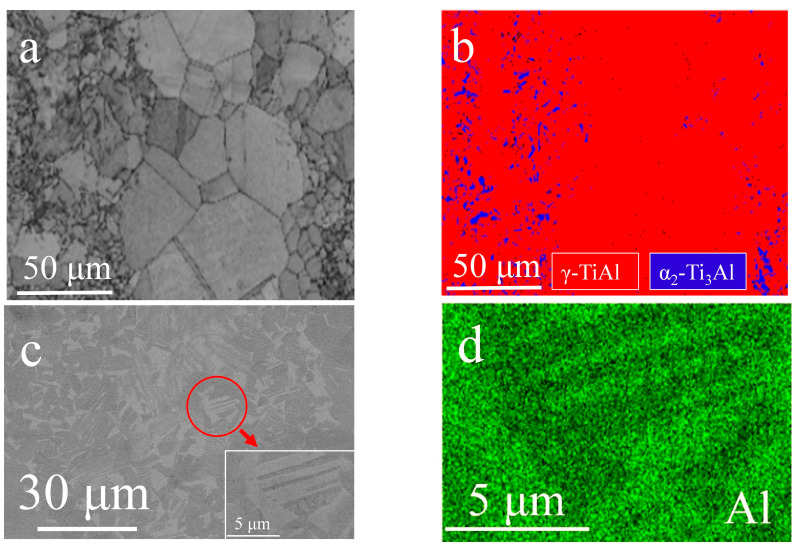

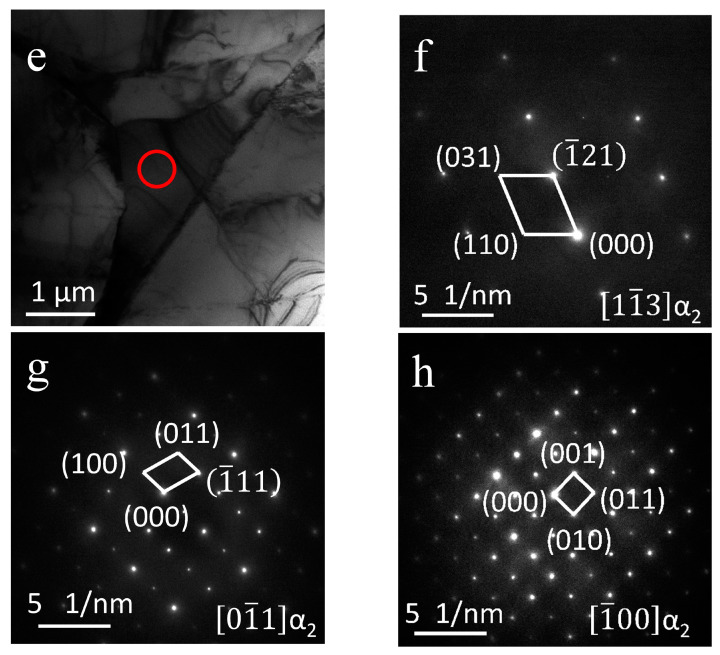
Microscopic analysis of the P–HIP Ti–48Al–3Nb–1.5Ta alloy: (**a**,**c**) Microstructures; (**b**) EBSD phase map of Figure 2a; (**d**) EDS mapping of Figure 2c; (**e**) TEM BF image; (**f**–**h**) SAED of the marked area of Figure 2e with different axes.

The P–HIP Ti–48Al–3Nb–1.5Ta alloy consists of 95.05% γ and 4.95% α_2_ phases, as represented in the EBSD phase map (Figure 2a,b), because the metastable α_2_ phase in the prealloyed powders almost completely transforms into the γ phase during the P–HIP process at 1200 °C, which occurs at the top of the single γ-phase area. The microstructures of the P–HIP Ti–48Al–3Nb–1.5Ta alloy were characterized mainly as near-gamma and locally duplex, as indicated in Figure 2c, consisting of γ (dark) and α_2_ (gray) phases with an average grain size of 5.9 μm, which is much more refined than those of the conventionally cast alloy as powder metallurgy is an effective method for refining grains. The EDS mapping (Figure 2d) of the magnified area of Figure 2c shows the α_2_ (Al-lean)/γ (Al-rich) lamella structure in the P–HIP Ti–48Al–3Nb–1.5Ta alloy. The TEM bright-field (BF) image (Figure 2e) and selected-area electron diffraction (SAED) patterns with different axes (Figure 2f–h) of the marked area in Figure 2e show the irregular α_2_ phase.

### 3.2. Characterization of the Surface Scale

**Figure 3:**
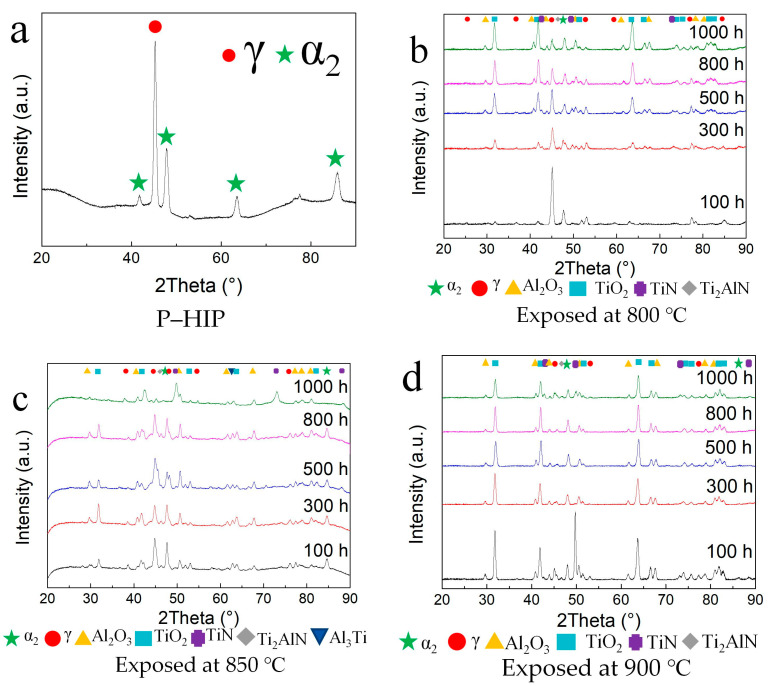
XRD patterns of the Ti–48Al–3Nb–1.5Ta (at. %) alloy: (**a**) P–HIP; (**b**) Exposed at 800 °C; (**c**) Exposed at 850 °C; (**d**) Exposed at 900 °C.

Because the type of surface scale after exposure determines the oxidation resistance, XRD analysis of the unsealed specimens’ surfaces was conducted to analyze the phase constitution, as shown in Figure 3. The XRD patterns and phase constitution were almost the same under different thermal-exposure conditions. α–Al_2_O_3_ and rutile–TiO_2_ were the primary components, and TiN and Ti_2_AlN were also detected. In addition, the patterns include γ and α_2_ phases, without a B2 phase detected as a peritectic TiAl [32], which are consistent with the P–HIP Ti–48Al–3Nb–1.5Ta alloy. The intensity of the γ and α_2_ phases decreases with increasing exposure temperature and duration. The peak of TiO_2_ becomes more prominent as the exposure duration increases at 800 °C, while notable peaks of TiO_2_ are discernible following thermal exposure at 850 °C and 900 °C for 100 h. A strong peak of TiN appears after exposure at 900 °C for 100h. However, as time passes, the TiN peak significantly weakens, indicating that TiN is generated rapidly at 900 °C and is subsequently covered by the continuously growing oxides of Ti and Al. No significant changes were observed in the α–Al_2_O_3_ peaks with variations in temperature and duration, indicating that α–Al_2_O_3_ is preferentially formable and relatively stable. Moreover, weak peaks of Ti_2_AlN were observed under different exposure conditions.

**Figure 4:**
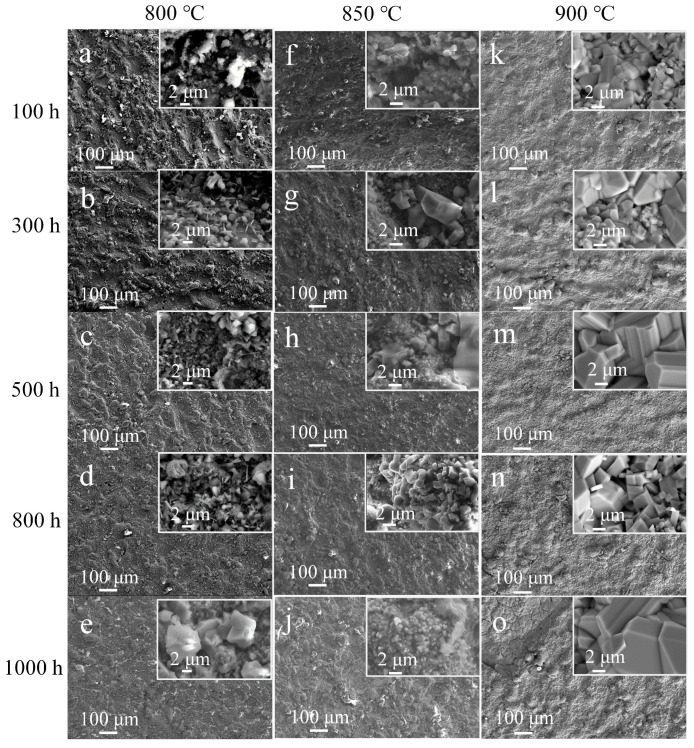
Surface morphology of the P–HIP Ti–48Al–3Nb–1.5Ta (at. %) alloy after thermal exposure to different conditions: (**a**) 800 °C, 100 h; (**b**) 800 °C, 300 h; (**c**) 800 °C, 500 h; (**d**) 800 °C, 800 h; (**e**) 800 °C, 1000 h; (**f**) 850 °C, 100 h; (**g**) 850 °C, 300 h; (**h**) 850 °C, 500 h; (**i**) 850 °C, 800 h; (**j**) 850 °C, 1000 h; (**k**) 900 °C, 100 h; (**l**) 900 °C, 300 h; (**m**) 900 °C, 500 h; (**n**) 900 °C, 800 h; (**o**) 900 °C, 1000 h.

**Figure 5:**
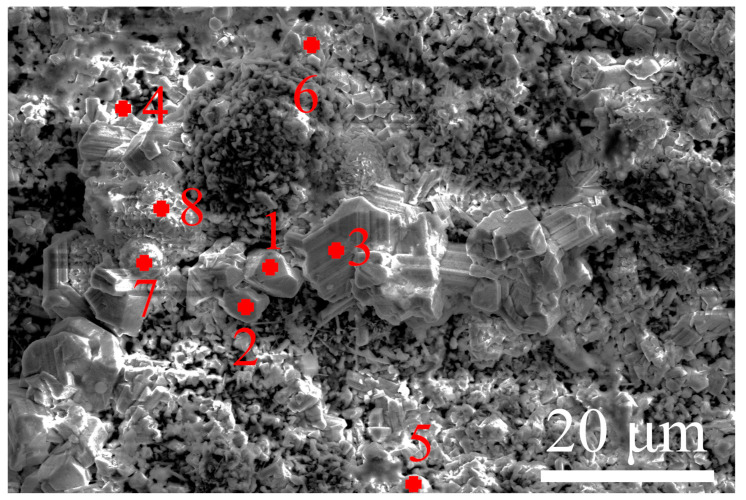
The surface morphology of the P–HIP Ti–48Al–3Nb–1.5Ta alloy exposed at 800 °C for 1000 h, with marks 1–8 indicating the EDS positions.

To investigate the scale characteristics, the surface morphologies of P–HIP Ti–48Al–3Nb–1.5Ta alloy after thermal exposure to different conditions were analyzed, as shown in Figure 4. The surface scale is mainly composed of irregularly shaped clusters and prismatic particles. EDS analysis was conducted on different surface areas of the specimen exposed at 800 °C for 1000 h, as shown in Figure 5 and Table 1. The results indicate that the surface scale is mainly composed of Ti, Al and O elements. There exists a higher content of Ti in prismatic particles (Locations 1, 2, 3, 4 and 6), while there exists a higher content of Al in irregularly shaped particles (Locations 7 and 8). Moreover, particles containing all Ti, Al, Nb and Ta elements are also observed (Location 5). According to the EDS and XRD results, the prismatic particles are TiO_2_, and the irregularly shaped particles are Al_2_O_3_. The surface morphologies change significantly with increasing exposure temperature and duration. Similarly fine and uneven scales were observed on the surface of the P–HIP Ti–48Al–3Nb–1.5Ta alloy exposed at 800 °C and 850 °C for 100 h, as shown in Figure 4a,f, and locally accumulated regions form at 850 °C. As the exposure duration increases, the surface scale becomes more continuous and accumulates, and TiO_2_ particles increase and grow. The surface of the Ti–48Al–3Nb–1.5Ta alloy exposed at 900 °C for 100 h displays an almost completely filled scale, as shown in Figure 4k, and TiO_2_ particles increase and grow significantly with increasing exposure duration, as shown in Figure 4o. The average size of the TiO_2_ particles increased from 1.74 μm to 4.02 μm while the maximum size increased from 4.96 μm to 12.70 μm, with the shape changing from equiaxed particles to elongated particles as the exposure duration extended from 100 h to 1000 h. A similar phenomenon has been reported in S. Taniguchi’s previous research [30]. As the TiO_2_ particles grow larger, fine TiO_2_ particles are generated between the large particles with gaps appearing, indicating that the oxidation resistance of the TiO_2_ layer is poor at high temperatures.

**Figure 6:**
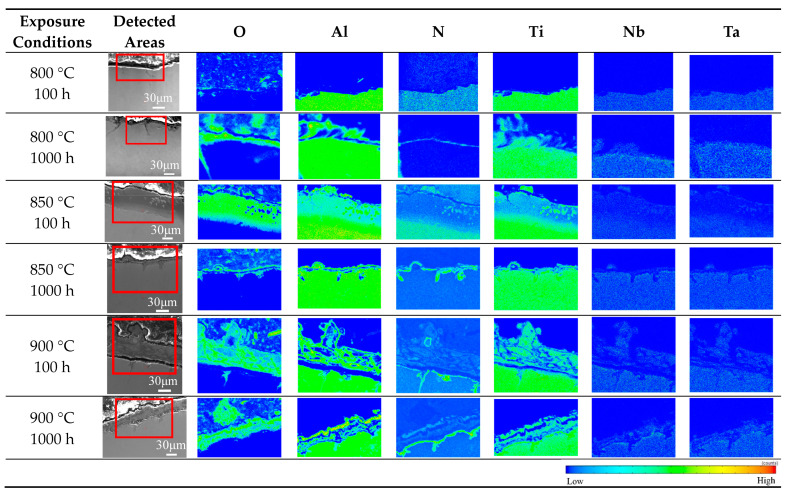
EPMA mapping on the near-surface of the P–HIP Ti–48Al–3Nb–1.5Ta alloy after exposure to different conditions, and the red squares in the column two show the detected areas.

In order to further analyze the distribution of different components of the surface scale after thermal exposure, EPMA mapping was performed on the near-surface of the specimens after thermal exposure at 800 °C, 850 °C and 900 °C for 100 h and 1000 h, respectively, as shown in Figure 6. The nitride layer is above the substrate and below the oxide layer. The oxidation of the TiAl alloy in air, broadly speaking, includes both oxidation and nitridation, and oxidation is usually superior to nitridation at a sufficiently high partial pressure of oxygen, which ensures the formation of a scale composed of TiO_2_ and Al_2_O_3_. When the oxide layer covers the entire surface of the substrate, further reactions are controlled by the inward diffusion of oxygen and nitrogen [45]. The oxidation layer exhibits a certain hindrance to the diffusion of these elements. Therefore, the activity of oxygen and nitrogen gradually decreases as the diffusion depth increases. Oxygen activity decreases faster because it is consumed preferentially. When the oxygen activity is lower than a certain critical value—that is, when the oxidation affected zone reaches a certain depth—it is conducive to the appearance of nitridation on the substrate. TiN is an excellent diffusion barrier that can prevent the diffusion of oxygen toward the substrate [46]. As a consequence, the typical structure of the scale on the exposed surface of P–HIP Ti–48Al–3Nb–1.5Ta alloy consists of external oxides and internal nitrides. The formation of the nitride sublayer below the oxide is significantly influenced by alloying elements such as Nb and Ta, which impede the diffusion of nitrogen toward the substrate [47].

The formation sequence and growth mode of oxides in the oxide layer are related to the free energy of formation and the activation energy of growth. The free energy of formation of the two oxides is highly negative at 800–900 °C; that is, they have a very low equilibrium decomposition pressure [48]. Therefore, TiAl alloys are prone to oxidation and form stable oxides in this temperature range [49]. According to the oxidation behavior of the TiAl alloy [12,50], Al_2_O_3_ is formed on the surface of the substrate during the initial stage of oxidation. With the formation of the Al_2_O_3_ layer, the Al on the surface of the TiAl substrate is gradually consumed, while Al diffuses slowly in the TiAl substrate. It is difficult to diffuse to the surface in a short time to replenish the consumed Al; thus, an Al-lean layer is formed below the thin layer of Al_2_O_3_, and the concentration of Ti in this layer gradually increases. When the Ti content reaches the critical value, it reacts with the inwardly diffused oxygen to form Ti oxides [51]. Common Ti oxides include TiO, Ti_2_O_3_ and TiO_2_, among which TiO_2_ has the best high-temperature stability [30]. The content of Al_2_O_3_ on the surface scale was higher than that of TiO_2_ after exposure at 850 °C for 100 h, as shown in Figure 4, and the thickness of the Al_2_O_3_ layer was greater than that of the TiO_2_ layer, as shown in Figure 6. However, the growth-activation energy of Al_2_O_3_ (502.4 kJ/mol) is higher than that of TiO_2_ (59.5 kJ/mol) [52], so the growth rate of TiO_2_ is much higher than that of Al_2_O_3_. With an increase in the exposure duration, TiO_2_ particles gradually cover the Al_2_O_3_ particles, resulting in a TiO_2_ layer formed on the Al_2_O_3_ layer [30], similar to the variation in surface morphologies shown in Figure 4.

Rutile TiO_2_ is a non-stoichiometric compound. Because of the large number of vacancy defects, this TiO_2_ layer allows for a fast diffusion rate of oxygen atoms, which represents a good path for oxygen penetration into the substrate [53]. Al_2_O_3_ is an ionically bonded compound with a dense structure, in which the diffusion rate of oxygen atoms is four orders of magnitude lower than that in the TiO_2_ layer. Therefore, once a continuous dense Al_2_O_3_ layer is formed, the diffusion of oxygen atoms almost terminates. Although the TiO_2_ layer cannot effectively prevent the oxidation of the substrate, it also reduces the oxygen concentration to a certain extent. Coupled with the obstructing effect of the Al_2_O_3_ layer, the oxygen concentration is further reduced, and the diffusion path of oxygen atoms becomes narrower and narrower, so the oxidation mass gain becomes slower and slower [30].

The addition of alloying elements can decrease the vacancy-formation energy of Al and increase that of Ti. As the Al vacancy concentration increases, it is easier for Al to diffuse to the surface and react with oxygen atoms, improving the oxidation resistance at high temperatures with a good Al_2_O_3_ protective film formed on the surface of the TiAl alloy [54]. Nb and Ti have the same valence electron structure and ionic radius, and they can be miscible in any proportion. Nb can occupy the normal Ti site. The replacement of Ti^4+^ with Nb^5+^ reduces the defect concentration of TiO_2_ as well as the diffusion rate of oxygen and metal ions in the oxide layer, inhibiting the formation and growth of TiO_2_ and facilitating the formation of a continuous dense Al_2_O_3_ layer on the surface of the TiAl alloy [24]. As the neighbor in the periodic table, Ta is similar to Nb, which can also reduce the oxygen solubility in the TiAl alloy and inhibit TiO_2_ growth [55]. Ta^5+^ replaces Ti^4+^ in the TiO_2_ lattice, resulting in an excess positive charge. The concentration of O^2-^ vacancies, which are responsible for O diffusion, decreases to offset this increase. Nb and Ta are both effective alloying elements that improve the oxidation resistance of TiAl alloys, and the oxidation rate at high temperatures of TiAl alloys containing Nb and Ta is significantly lower than that of simple binary TiAl alloys with the same Al content [47]. There are very small amounts of Nb and Ta in the oxide layer above the nitride layer, as we can see from the 900 °C EPMA mapping results in Figure 6, whereas there is no such phenomenon at 800 °C and 850 °C, because the ability of Nb and Ta to diffuse toward the substrate surface increases with increasing temperature. Because there is no diffraction peak for oxides of Ta or Nb in the XRD patterns, it is assumed that they replace Ti in the rutile lattice [31], thus impeding the oxidation process at higher temperatures.

**Figure 7:**
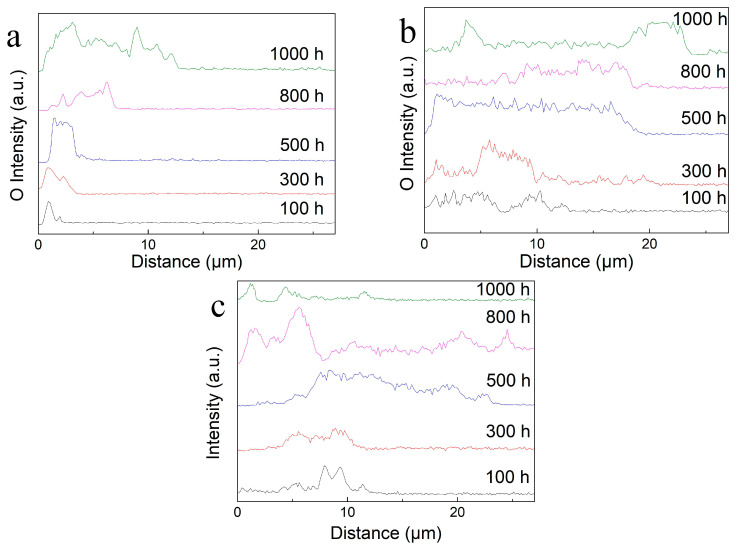
Oxygen contents vary with the depth of different sample surfaces after exposure: (**a**) At 800 °C; (**b**) At 850 °C; (**c**) At 900 °C.

The variation in the oxide-layer thickness after thermal exposure was further analyzed using EDS line scanning, as shown in Figure 7. With increasing thermal exposure temperature and duration, the oxide-layer thickness gradually increases at a slow rate. The thicknesses of the oxide layer are about 12.7 μm and 23.7 μm after thermal exposure for 1000 h at 800 °C and 850 °C, respectively, which is smaller than 24.5 μm, the thickness of a Ti–47.5Al–2.5V–1.0Cr–0.2Zr (at %) oxide layer after exposure at 750 °C for 300 h [56], indicating superior oxidation resistance. The thickness of the oxide layer is about 25.2 μm after thermal exposure at 900 °C for 800 h, and the decrease in the oxide-layer thickness after 1000 h may be related to the spalling of the oxide layer.

In general, the oxide layer becomes significantly continuous with increasing exposure temperature, especially for short exposure durations. However, after exposure for 1000 h, the surface morphologies all show a continuous and fully filled scale, similar to that in a previous study [57].

### 3.3. Microstructure Stability

**Figure 8:**
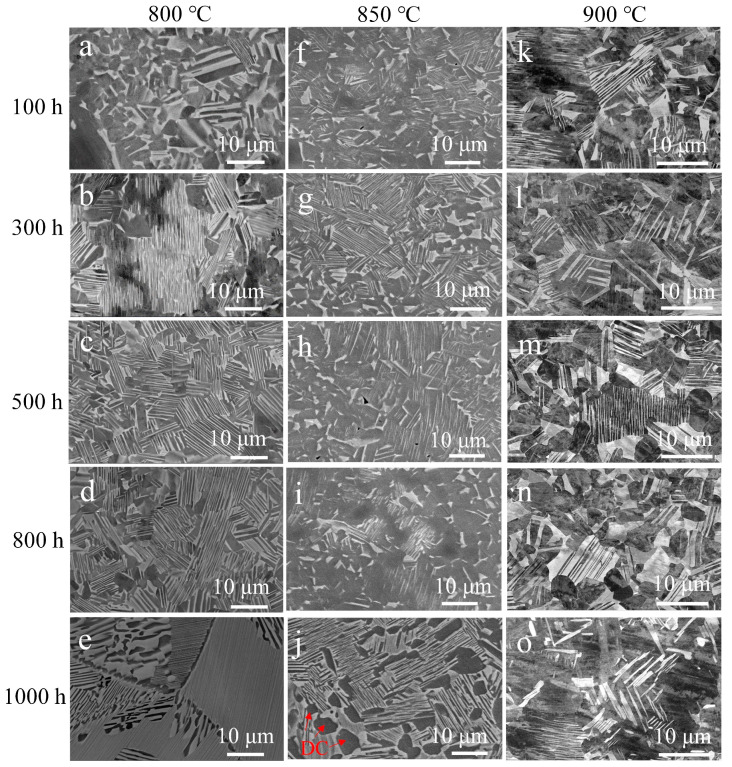
Microstructures of the Ti–48Al–3Nb–1.5Ta alloy after exposure to different conditions: (**a**) 800 °C, 100 h; (**b**) 800 °C, 300 h; (**c**) 800 °C, 500 h; (**d**) 800 °C, 800 h; (**e**) 800 °C, 1000 h; (**f**) 850 °C, 100 h; (**g**) 850 °C, 300 h; (**h**) 850 °C, 500 h; (**i**) 850 °C, 800 h; (**j**) 850 °C, 1000 h; (**k**) 900 °C, 100 h; (**l**) 900 °C, 300 h; (**m**) 900 °C, 500 h; (**n**) 900 °C, 800 h; (**o**) 900 °C, 1000 h.

**Figure 9:**
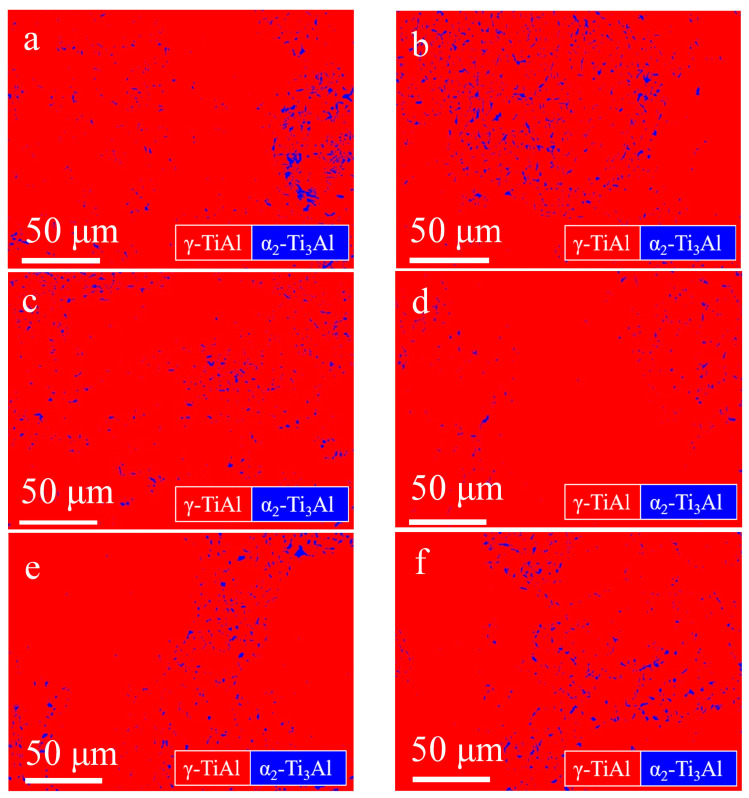
EBSD phase constitutions of the Ti–48Al–3Nb–1.5Ta alloy after exposure to different conditions: (**a**) 800 °C, 100 h; (**b**) 800 °C, 1000 h; (**c**) 850 °C, 100 h; (**d**) 850 °C, 1000 h; (**e**) 900 °C, 100 h; (**f**) 900 °C, 1000 h.

**Figure 10:**
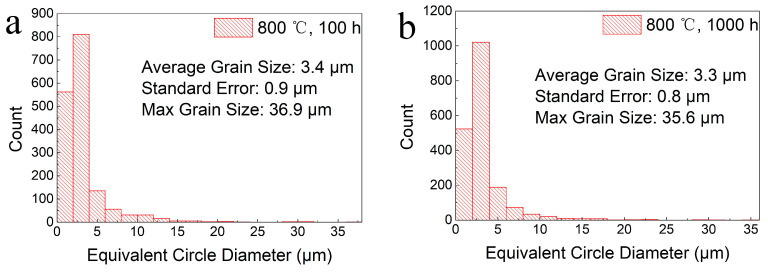

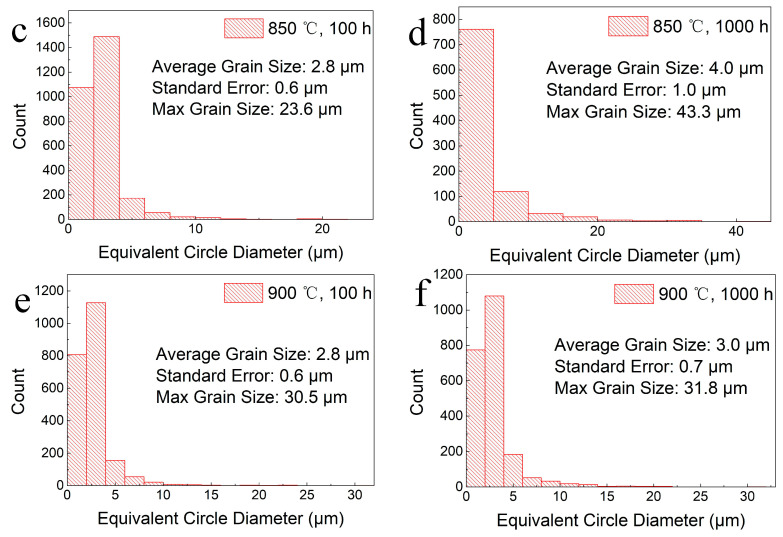
Grain size distributions of the Ti–48Al–3Nb–1.5Ta alloy after exposure to different conditions: (**a**) 800 °C, 100 h; (**b**) 800 °C, 1000 h; (**c**) 850 °C, 100 h; (**d**) 850 °C, 1000 h; (**e**) 900 °C, 100 h; (**f**) 900 °C, 1000 h.

In order to analyze the microstructural evolution of the P–HIP Ti–48Al–3Nb–1.5Ta alloy during thermal exposure, BSE-SEM microstructure morphology analysis and EBSD phase-composition analysis were performed on the internal cross-sections of the sealed specimens after exposure to different conditions, as shown in Figure 8 and Figure 9. The dark region is the γ phase, whereas the white region is the α_2_ phase in Figure 8. The phase composition and grain-size distribution analyzed using EBSD are shown in Table 2 and Figure 10. The results reveal that the grain size does not grow significantly after thermal exposure to different conditions, and the γ phase increases slightly, less than 3.5%, compared with P–HIP, as depicted in Section 3.1. The increase in the γ-phase volume fraction is caused by the α_2_-phase dissolution at 800–900 °C, and longitudinal and transverse dissolution leads to thinning and fracture of the α_2_ lamella [58,59]. At the same time, with the extension of the thermal exposure duration, the DC of the γ phase is observed near the grain boundaries due to the reduction of lamella near the grain boundaries, which expands the γ phase region, as marked in the microstructure after exposure at 850 °C for 1000 h in Figure 8.

Although the γ lamella and the α_2_ lamella have an interphase distribution, the adjacent interfaces are not parallel to each other, and the shape of the γ lamella and α_2_ is probably irregular or even curved. The thicknesses of different γ lamellae vary greatly, and the γ lamella is not regular but is like a wedge embedded within α_2_ lamellae. In addition, there is a great difference in the thicknesses of different α_2_ lamellae. The widest reaches 2.4 μm, while the narrowest is only 120 nm in the P–HIP Ti–48Al–3Nb–1.5Ta alloy, as shown in Figure 2c. This difference leads to a high content of α_2_ lamella in local areas, resulting in a state of agglomeration and decomposition during thermal exposure. The α_2_ lamella is metastable, leading to a tendency to transform into the most stable form during long-term exposure at high temperatures. Therefore, the metastable α_2_ lamella decomposes into a fine α_2_ + γ lamella [60], and the newly generated γ/α_2_ interface is parallel to the original α_2_ lamella, which is called “parallel decomposition” [9].

To reduce the total free energy of the system under long-term high temperatures, three types of microstructural evolutions may be generated for the lamella structure of the TiAl alloy: (1) phase transformations, (2) continuous coarsening, and (3) discontinuous coarsening. The phase transformation is manifested as the dissolution of the α_2_ lamella, causing the volume fraction and composition of the γ and α_2_ phases to change toward the equilibrium volume fraction and phase composition determined by the Ti-Al binary-phase diagram at this temperature [61], reducing the chemical free energy of the system [62]. Since α_2_/γ is a low-energy semi-coherent flat interface in the lamella structure of the TiAl alloy and satisfies the Blackburn orientation relation, i.e., ${\left\{111\right\}}_{\gamma }∥{\left\{0001\right\}}_{\alpha_{2}}$ and ${<11\bar{2}0>}_{\alpha_{2}}∥{<110>}_{\gamma }$, there is no obvious interfacial curvature. Therefore, continuous coarsening within the lamella is relatively difficult. However, the lamella structure has poor thermal stability and is prone to DC with grain boundary migration because of the existence of steps between α_2_ and γ lamellae at the grain boundaries [63].

**Figure 11:**
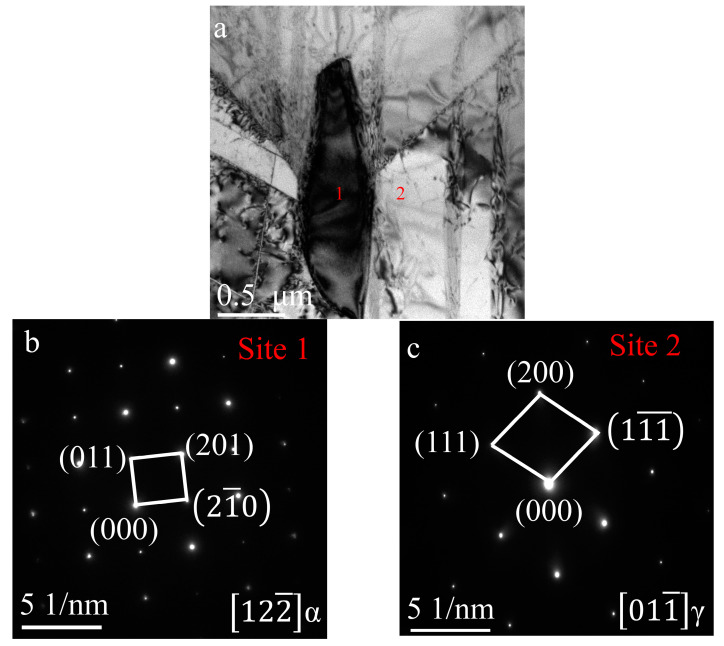

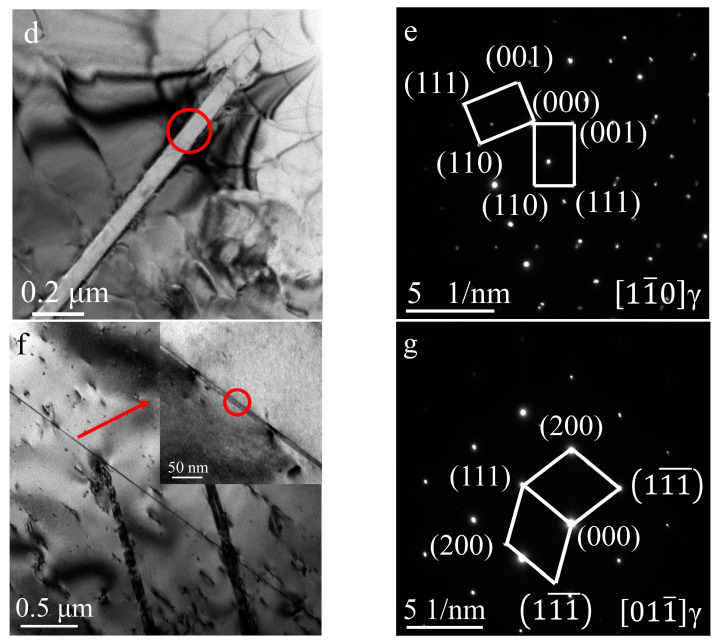
TEM images of the P–HIP Ti–48Al–3Nb–1.5Ta alloy exposed at 800 °C for 1000 h: (**a**) BF image showing two phases; (**b**) SAED of site 1 in Figure 11a; (**c**) SAED of site 2 in Figure 11a; (**d**) BF image of γ-phase twins; (**e**) SAED of circled area in Figure 11d; (**f**) BF image of nanotwins; (**g**) SAED of circled area in Figure 11f.

The specimens exposed at 800 °C for 1000 h were analyzed using TEM, and the BF images, SAED and EDS are shown in Figure 11 and Table 3. It can be seen that the matrix consists of a γ phase, the massive precipitate near the grain boundaries has an α_2_ phase, and there are γ-phase twins. In addition, the nanotwins can be seen from the BF images and SAED in Figure 11f,g. The 6 nm average width of these nanotwins is the result of plastic deformation during the P–HIP process at 150 MPa pressure, and it remained so after thermal exposure because the annealing twins’ width is in the micro dimension [64].

### 3.4. Mechanical Performance

**Figure 12:**
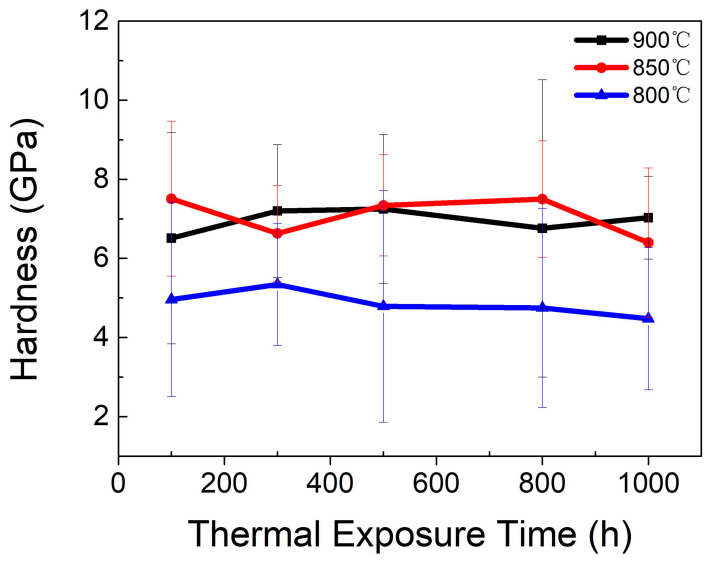
Hardness of the Ti–48Al–3Nb–1.5Ta alloy after exposure to different conditions.

To investigate the effects of thermal exposure on the mechanical performance of the P–HIP Ti–48Al–3Nb–1.5Ta alloy, nanoindentation analysis was performed on specimens after thermal exposure to different conditions, as shown in Figure 12. The hardness after thermal exposure at 850 and 900 °C increases by about 2 GPa compared with the hardness of the P–HIP state, which is 4.6 GPa. This is mainly due to the O solution during thermal exposure, which causes lattice distortion of the matrix and the formation of new phases, resulting in surface hardening of the alloy [65]. The hardness after thermal exposure at 850 °C and 900 °C is higher than that at 800 °C, indicating that oxidation is more serious at higher temperatures. With the extension of thermal exposure duration, the hardness fluctuates in a narrow range and tends to decrease to different degrees, which is related to the equiaxed γ grains increasing and growing continuously, the α_2_ phase decreasing and the lamella coarsening [14]. The strength of the TiAl alloy is inversely proportional to the grain size following the Hall–Petch formula. Therefore, the α_2_ + γ lamella coarsening and equiaxed γ growth during thermal exposure reduce the hardness and tensile properties of the alloy. The lamella spacing is negatively correlated with the hardness of the nanoindentation; that is, the larger the lamella spacing, the smaller the hardness of the nanoindentation. When the indenter is pressed into the matrix, plastic deformation occurs near the indenter, and elastic deformation occurs in the area away from the indenter. The main mechanism of plastic deformation is dislocation slip. When the γ/α_2_ lamella spacing is small, the dislocation slip encounters more γ/α_2_ boundaries, resulting in higher hardness values. During thermal exposure at 800–900 °C, a phase transformation from α_2_ to γ will occur, and the hardness of the γ phase is less than that of the α_2_ phase, so there will be a trend of hardness decline with the extension of thermal exposure duration.

**Figure 13:**
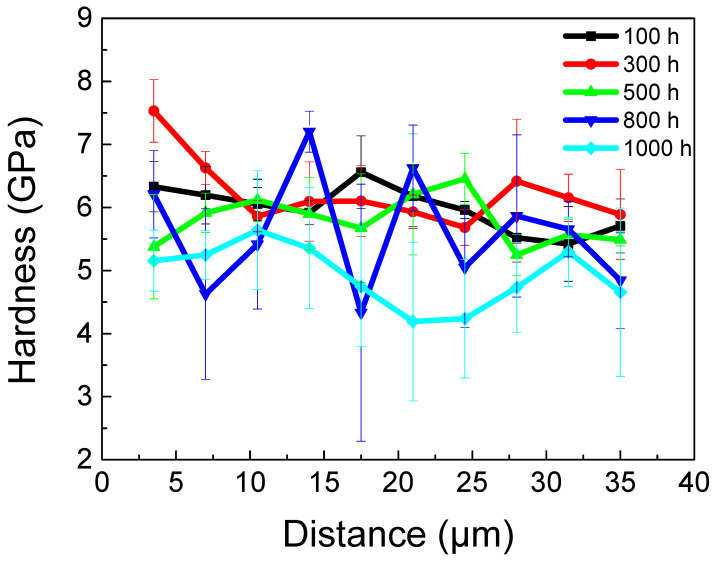
Hardness variation along the depth direction from the edge of the specimens after exposure at 800 °C for different durations.

To clarify the variation rule of hardness in the near-surface region of the P–HIP Ti–48Al–3Nb–1.5Ta alloy along the depth direction after thermal exposure at 800 °C for different durations, nanoindentation tests were conducted along the depth direction from the edge of the unsealed specimens. The hardness results are shown in Figure 13. From the results of thermal exposure for 100 h, 300 h and 500 h, it can be seen that the hardness decreases with increasing depth from the specimen surface, since an O solution on the surface of the alloy leads to surface hardening. For titanium alloys, the relationship between hardness and surface O concentration can be expressed as follows [66]:

(1) $$H=H_{0}+b{\left(x_{O}\right)}^{\frac{1}{2}}$$

where H represents the hardness at the specified position, *H*_0_ represents the hardness of the matrix, b is a constant, and *x_O_* represents the O infiltration concentration at the specified position. As the O concentration gradually decreases from the surface along the depth direction, the hardness gradually decreases accordingly. In addition, an “influence zone” with an increased α_2_ volume fraction is formed below the oxide layer in the TiAl alloy, the hardness of which is higher than that of the matrix, and the hardness gradually decreases from the edge to the matrix [66]. After thermal exposure for 800 h, the hardness values fluctuated greatly, which may be due to lamella coarsening and grain growth, and the test indenter easily fell into different phases with large differences in hardness. According to the nanoindentation hardness of the PST–TiAl alloy [67], the hardness of γ phase nanoindentation is 5.2 ± 0.1 GPa, and that of α_2_ phase nanoindentation is 7.4 ± 0.5 GPa. It should be noted that the low hardness values after exposure for 1000 h may be related to the local spallation of the oxide layer.

**Figure 14:**
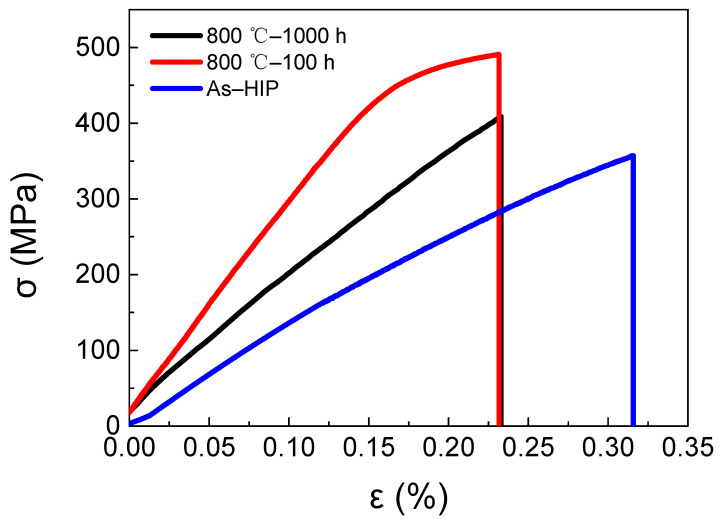
Tensile stress–strain curve of P–HIP after exposure at 800 °C for 100 and 1000 h.

**Figure 15:**
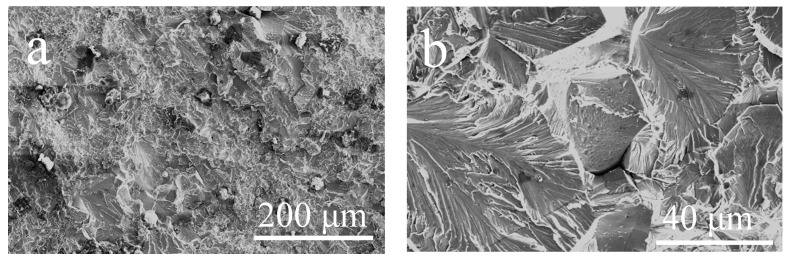
SEM images of the fracture surfaces after exposure at 800 °C for 1000 h: (**a**) Low magnification; (**b**) High magnification.

Tensile tests at room temperature were performed on specimens exposed at 800 °C for 100 and 1000 h, respectively. The stress–strain curves together with the result of the P–HIP Ti–48Al–3Nb–1.5Ta alloy are shown in Figure 14. After thermal exposure at 800 °C for 100 and 1000 h, the tensile strengths were 491 MPa and 409 MPa, respectively, and the fracture strain was 0.23%. The fracture morphology of the tensile specimen exposed at 800 °C for 1000 h is shown in Figure 15, which indicates a typical brittle cleavage section, as expected. Nanotwins, as shown in Figure 11, are a special type of plane defect that can greatly hinder the dislocation movement and enhance the strength of alloys, particularly when the slip plane and Burgers vectors are not aligned with the twin boundaries [68]. Compared with the P–HIP Ti–48Al–3Nb–1.5Ta alloy, whose tensile strength is 357.09 MPa and whose fracture strain is 0.32%, the strength increases by more than 50 MPa and the fracture strain decreases by about 0.1% after the thermal exposure. Although the fracture strain of this TiAl alloy is marginally less than that reported in conventional TiAl alloys, preliminary results have shown that the microstructure and properties of the alloy can be optimized through heat treatments to increase its plasticity and to meet application requirements. The excellent oxidation resistance of this alloy is the primary topic of this study; subsequent studies will focus on the modification of the microstructure and characteristics by heat treatment.

The increase in strength after thermal exposure may be due to the increased oxygen content of the alloy [69]. In addition, during thermal exposure at 800 °C, α_2_ → γ phase transformation occurs, γ grains precipitate in the α_2_ + γ lamellae, and this parallel decomposition in the lamella makes the lamella refined to a certain extent. The thin lamella provides more obstacles to dislocations and twins, and it reduces internal stress by reducing the length of dislocation accumulation at the interface, thereby increasing resistance to cross-lamella deformation, resulting in higher tensile strength after thermal exposure at 800 °C for 100 h and 1000 h compared with the P–HIP state [9]. The tensile strength after thermal exposure for 1000 h was lower than that after thermal exposure for 100 h, mainly because of the influence of grain-size growth, according to the Hall–Patch formula. The appearance and stepwise growth of the Al-lean layer during thermal exposure will lead to the formation of a brittle layer on the surface, resulting in a decrease in the tensile fracture strain at room temperature [70]. In addition, the decrease in tensile fracture strain at room temperature after thermal exposure may also be related to the residual stress on the surface [8]. Moreover, it has been shown that the instability of the lamella colony also causes the ductility of the alloy to deteriorate, and during long-term exposure, the fracture strain decreases because of the decomposition of α_2_ lamellae and the merger of adjacent γ lamellae [9].

## 4. Conclusions

The Ti–48Al–3Nb–1.5Ta alloy was prepared by powder hot isostatic pressing (P–HIP) at 1200 °C with 150 MPa for 4 h and exposed at 800 °C, 850 °C and 900 °C for 100 h, 300 h, 500 h, 800 h and 1000 h. The surface-scale characterization, microstructure stability and mechanical performance of the P–HIP Ti–48Al–3Nb–1.5Ta alloy after thermal exposure were investigated and clarified. The main conclusions are as follows:

1. The surface scale is composed of oxides and nitrides, mainly Al_2_O_3_, TiO_2_, TiN and Ti_2_AlN; among these, Al_2_O_3_ is preferentially generated and then covered by rapidly growing TiO_2_ as the thermal exposure duration increases. The nitrides appear later than the oxides and exist between the oxide layer and the substrate. With increasing thermal exposure temperature and duration, the surface scale becomes more continuous, the prismatic TiO_2_ particles grow larger, and the oxide layer thickens. The average size of the TiO_2_ particles increased from 1.74 μm to 4.02 μm while the maximum size increased from 4.96 μm to 12.70 μm, with the shape changing from equiaxed particles to elongated particles, as the exposure duration extended from 100 h to 1000 h at the exposure temperature of 900 °C;
2. The addition of Ta and Nb can improve the oxidation resistance of TiAl alloys, and the oxidation resistance of Ti–48Al–3Nb–1.5Ta alloy is superior to that of the Ti–47.5Al–2.5V–1.0Cr–0.2Zr alloy, because Ta^5+^ and Nb^5+^ replace Ti^4+^ in the rutile lattice and weaken O diffusion, thus impeding the oxidation process;
3. Compared with the P–HIP Ti–48Al–3Nb–1.5Ta alloy, the grain size does not increase significantly, and the γ phase increases slightly (less than 3%) with the decomposition of the α_2_ phase after thermal exposure. With increasing thermal exposure duration, the γ phase initiates discontinuous coarsening (DC) due to the reduction in lamellae near the grain boundaries, which expands the γ-phase region;
4. Compared with the P–HIP Ti–48Al–3Nb–1.5Ta alloy, the hardness increases by about 2 GPa, the tensile strength increases by more than 50 MPa and the fracture strain decreases by about 0.1% after thermal exposure. With the extension of the thermal exposure duration, the hardness tends to decrease, because of the equiaxed γ grains increasing and growing continuously, the α_2_ phase decreasing and the lamellae coarsening. As the depth increases from the surface of the P–HIP Ti–48Al–3Nb–1.5Ta alloy after thermal exposure, the hardness decreases overall.

The P–HIP Ti–48Al–3Nb–1.5Ta alloy, a potential low-density alloy, exhibits attractive microstructure stability and does not significantly lose mechanical properties after thermal exposure at 800–900 °C for up to 1000 h. This study will provide beneficial guidance for high temperature applications involving weight reduction. Future research will focus on how heat treatments can improve the alloy’s microstructure and plasticity.

## Figures and Tables

**Table 1:** EDS analysis results of the P–HIP Ti–48Al–3Nb–1.5Ta alloy (at. %) exposed at 800 °C for 1000 h shown in Figure 5.

Position	Ti	Al	O	Nb	Ta	C
1	27.57	-	68.71	-	-	3.72
2	39.58	0.43	56.89	-	-	3.11
3	27.61	-	68.21	-	-	4.18
4	33.13	5.86	61.01	-	-	-
5	17.43	7.35	48.23	0.99	0.43	25.58
6	26.34	0.72	69.68	-	-	3.25
7	6.50	26.73	66.78	-	-	-
8	2.67	25.89	58.14	-	-	13.30

**Table 2:** EBSD phase constitution of the Ti–48Al–3Nb–1.5Ta alloy after thermal exposure to different conditions.

Temperature (°C)	Duration (h)	γ Phase (%)	α_2_ Phase (%)
800	100	$96.9_{-0.15}^{+0.33}$	$3.1_{-0.17}^{+0.58}$
	1000	$95.5_{-0.09}^{+0.26}$	$4.5_{-0.21}^{+0.39}$
850	100	$97.5_{-0.36}^{+0.25}$	$2.5_{-0.61}^{+0.77}$
	1000	$98.5_{-0.11}^{+0.31}$	$1.5_{-0.46}^{+0.42}$
900	100	$97.5_{-0.29}^{+0.15}$	$2.5_{-0.42}^{+0.61}$
	1000	$97.5_{-0.30}^{+0.11}$	$2.5_{-0.55}^{+0.43}$

**Table 3:** EDS results of site 1 and site 2 in Figure 11a (at. %).

Element	Al	Ti	Nb	Ta
1	31.62	65.32	1.53	1.50
2	49.88	45.61	2.94	1.55

## Data Availability

All the data generated during this study are included in this article.

## References

[1] Appel H.F., Paul J., Oehring M. (2011). Gamma Titanium Aluminide Alloys Science and Technology.

[2] Kim Y., Kim S. (2018). Advances in Gammalloy Materials–Processes–Application Technology: Successes, Dilemmas, and Future. Jom.

[3] Kothari K., Radhakrishnan R., Wereley N.M. (2012). Advances in gamma titanium aluminides and their manufacturing techniques. Prog. Aerosp. Sci..

[4] Chandran A., Ganesan H., Cyron C.J. (2024). Studying the effects of Nb on high-temperature deformation in TiAl alloys using atomistic simulations. Mater. Des..

[5] Guo R., Xiong G., Liu J., Wang Y., Davoodi D., Miri R., Tayebi M. (2023). Tribological behavior of Ti-Al-Nb alloy with different Ta additions for high temperature applications. Mater. Lett..

[6] Chen X., Tang B., Wei B., Zhang X., Li J. (2023). Investigation on recrystallization behavior of Ti-47Al-1.5Re-X (Cr, Mn, V, Nb) alloy during hot deformation. Mater. Lett..

[7] Hu R., Wang X., Yang J., Fu H. (2017). Microstructures Thermal Stability and Evolution Mechanism of TiAl-Base Alloys and Their Effects on Mechanical Properties. Aerosp. Sci. Technol..

[8] Pather R., Mitten W.A., Holdway P., Ubhi H., Wisbey A., Brooks J. (2003). The effect of high temperature exposure on the tensile properties of γ TiAl alloys. Intermetallics.

[9] Huang Z.W., Cong T. (2010). Microstructural instability and embrittlement behaviour of an Al-lean, high-Nb γ-TiAl-based alloy subjected to a long-term thermal exposure in air. Intermetallics.

[10] Huang Z.W., Voice W., Bowen P. (2000). The effects of long-term air exposure on the stability of lamellar TiAl alloys. Intermetallics.

[11] Huang Z.W. (2013). Thermal stability of Ti–44Al–4Nb–4Zr–0.2Si–1B alloy. Intermetallics.

[12] Zhou L.Z., Lupinc V., Guo J.T. (2003). Microstructural stability of the intermetallic Ti–45Al–2W–0.5Si–0.5B in the 800–980 °C temperature range. Mater. Sci. Eng. A.

[13] Seo D.Y., Zhao L., Beddoes J. (2002). Microstructural evolution during heat treatments in Ti-45 and 47Al–2Nb–2Mn + 0.8vol.%TiB2 XDTM alloys. Mater. Sci. Eng. A.

[14] Leyens C., Braun R., Frohlich M., Hovsepian P. (2006). Recent progress in the coating protection of gamma titanium-aluminides. Jom.

[15] Pan Y., Han D., Huang S., Niu Y., Liang B., Zheng X. (2023). Thermal insulation performance and thermal shock resistance of plasma-sprayed TiAlCrY/Gd_2_Zr_2_O_7_ thermal barrier coating on γ-TiAl alloy. Surf. Coat. Technol..

[16] Zhang K., Xin L., Ma T., Chang H., Lu Y., Feng C., Zhu S., Wang F. (2022). Investigation of the role of silicon in TiAlSiN coating deposited on TiAl alloys during long-term oxidation. Corros. Sci..

[17] Haanappel V.A.C., Clemens H., Stroosnijder M.F. (2002). The high temperature oxidation behaviour of high and low alloyed TiAl-based intermetallics. Intermetallics.

[18] Xiang L.L., Zhao L.L., Wang Y.L., Zhang L., Lin J. (2012). Synergistic effect of Y and Nb on the high temperature oxidation resistance of high Nb containing TiAl alloys. Intermetallics.

[19] Hadi M., Bayat O., Meratian M., Shafyer A., Ebrahimzadeh I. (2018). Oxidation Properties of a Beta-Stabilized TiAl Alloy Modified by Rare Earth Elements. Oxid. Met..

[20] Du H.L., Aljarany A., Datta P.K., Burnell J. (2005). Oxidation behaviour of Ti–46.7Al–1.9W–0.5Si in air and Ar–20%O_2_ between 750 and 950 °C. Corros. Sci..

[21] Pilone D., Felli F. (2012). Isothermal oxidation behaviour of TiAl–Cr–Nb–B alloys produced by induction melting. Intermetallics.

[22] Wendler B.G., Kaczmarek Ł. (2005). Oxidation resistance of nanocrystalline microalloyed γ-TiAl coatings under isothermal conditions and thermal fatigue. J. Mater. Process. Technol..

[23] Haanappel V.A.C., Clemens H., Stroosnijder M.F. (2002). The effect of microstructure on the oxidation behaviour of Ti-46.5Al-4(Cr,Nb,Ta,B) and Ti-47 Al-4(Cr,Nb,Mo,B). Mater. High Temp..

[24] Przybylski K., Prazuch J., Brylewski T., Durda E. (2013). High Temperature Oxidation Behaviour of Tial8Nb Alloy. Arch. Metall. Mater..

[25] Mengis L., Ulrich A.S., Watermeyer P., Liebscher C., Galetz M. (2021). Oxidation behaviour and related microstructural changes of two β0–phase containing TiAl alloys between 600 °C and 900 °C. Corros. Sci..

[26] Banumathy S., Sruti Neelam N., Chandravanshi V., Bhattacharjee A., Ravi K. (2018). The Effect of Nb addition on microstructure, oxidation behavior and strength of some γ-TiAl alloys. Mater. Today Proc..

[27] Małecka J. (2022). Resistance to High-Temperature Oxidation of Ti-Al-Nb Alloys. Materials.

[28] Mitoraj M., Godlewska E., Heintz O., Geoffroy N., Fontana S., Chevalier S. (2011). Scale composition and oxidation mechanism of the Ti–46Al–8Nb alloy in air at 700 and 800 °C. Intermetallics.

[29] Fergus J.W. (2002). Review of the effect of alloy composition on the growth rates of scales formed during oxidation of gamma titanium aluminide alloys. Mater. Sci. Eng. A.

[30] Li X. (2014). Research on Microstructure and Mechanical Properties of Ti-45Al-10Nb Alloy Prepared by Powder Metallurgy. Ph.D. Thesis.

[31] Pfeiler M., Scheu C., Hutter H., Schnöller J., Michotte C., Mitterer C., Kathrein M. (2009). On the effect of Ta on improved oxidation resistance of Ti–Al–Ta–N coatings. J. Vac. Sci. Technol. A.

[32] Pan H., Zhang K., Chen J., Zhang X., Zhao H., Hu R. (2023). Variant selection of massive γ subgrains and corresponding mechanical properties in Ta containing γ-TiAl-based alloys. Mater. Lett..

[33] Chen S., Tan Y., Wang X., Cao F., Wang L., Su Y., Guo J. (2023). Individual and synergistic effects of Ta and Mn on optimizing the microstructures and mechanical properties of TiB reinforced high Nb–TiAl composites. J. Mater. Res. Technol..

[34] Liu Z., Wang C., Wang W., Xu G., Liu X. (2021). Effects of Tantalum on the microstructure and properties of Ti-48Al-2Cr-2Nb alloy fabricated via laser additive manufacturing. Mater. Charact..

[35] Gao Z., Hu R., Huang Z., Wu Y., Li J., Zhou M. (2022). Metastable transformation behavior in a Ta-containing TiAl-Nb alloy during continuous cooling. J. Alloy Compd..

[36] Zhang K., Hu R., Lei T., Yang J. (2019). Refinement of massive γ phase with enhanced properties in a Ta containing γ-TiAl-based alloys. Scr. Mater..

[37] Zhang K., Hu R., Li J., Yang J., Gao Z. (2020). Grain refinement of 1 at.% Ta-containing cast TiAl-based alloy by cyclic air-cooling heat treatment. Mater. Lett..

[38] Hu D., Huang A.J., Wu X. (2007). On the massive phase transformation regime in TiAl alloys: The alloying effect on massive/lamellar competition. Intermetallics.

[39] Lapin J., Pelachová T., Dománková M. (2018). Long-term creep behaviour of cast TiAl-Ta alloy. Intermetallics.

[40] Saage H., Huang A.J., Hu D., Loretto M.H., Wu X. (2009). Microstructures and tensile properties of massively transformed and aged Ti_46_Al_8_Nb and Ti_46_Al_8_Ta alloys. Intermetallics.

[41] Loretto M.H., Wu Z., Chu M.Q., Saage H., Hu D., Attallah M.M. (2012). Deformation of microstructurally refined cast Ti_46_Al_8_Nb and Ti_46_Al_8_Ta. Intermetallics.

[42] Lapin J., Gabalcová Z. (2011). Solidification behaviour of TiAl-based alloys studied by directional solidification technique. Intermetallics.

[43] Yan M., Yang F., Lu B., Chen C., Sui Y., Guo Z. (2021). Microstructure and Mechanical Properties of High Relative Density γ-TiAl Alloy Using Irregular Pre-Alloyed Powder. Metals.

[44] (2021). Metallic materials—Tensile testing—Part 1: Method of test at room temperature.

[45] Bik M., Galetz M., Mengis L., White E., Wieczorek W., Łyszczarz K., Mroczka K., Marchewka J., Sitarz M. (2023). Oxidation behaviour of uncoated and PDC-SiAlOC glass-coated TiAl at 750 °C in dry and humid air. Appl. Surf. Sci..

[46] Pérez P. (2005). Influence of nitriding on the oxidation behaviour of titanium alloys at 700 °C. Surf. Coat. Technol..

[47] Vojtěch D., Popela T., Kubásek J., Maixner J., Novák P. (2011). Comparison of Nb- and Ta-effectiveness for improvement of the cyclic oxidation resistance of TiAl-based intermetallics. Intermetallics.

[48] Kovács K., Perczel I.V., Josepovits V.K., Kiss G., Réti F., Deák P. (2002). In situ surface analytical investigation of the thermal oxidation of Ti–Al intermetallics up to 1000 °C. Appl. Surf. Sci..

[49] Gale W.F., Totemeier T.C., Gale W.F., Totemeier T.C. (2004). 8—Thermochemical data. Smithells Metals Reference Book.

[50] Dudziak T., Rząd E., Morgiel J., Wytrwal-Sarna M., Kirchner A., Pomorska M., Boron L., Polczyk T., Moskal G., Toboła D. (2022). Scale mass gain, morphology and phase composition of air and steam oxidized electron beam melted and cast Ti–48Al–2Nb–0.7Cr–0.3Si alloys. Intermetallics.

[51] Pan Y., Yang Y., Zhou Q., Qu X., Cao P., Lu X. (2024). Achieving synergy of strength and ductility in powder metallurgy commercially pure titanium by a unique oxygen scavenger. Acta Mater..

[52] Taylor T.N., Paffett M.T., Whang S.H., Pope D.P., Liu C.T. (1992). Oxide properties of a γ-TiAl: A surface science study. High Temperature Aluminides and Intermetallics.

[53] Huang H., Yan H., Duan M., Ji J., Liu X., Jiang H., Liu B., Sajid S., Cui P., Li Y. (2021). TiO_2_ surface oxygen vacancy passivation towards mitigated interfacial lattice distortion and efficient perovskite solar cell. Appl. Surf. Sci..

[54] Ping F., Hu Q., Bakulin A.V., Kulkova S.E., Yang R. (2016). Alloying effects on properties of Al_2_O_3_ and TiO_2_ in connection with oxidation resistance of TiAl. Intermetallics.

[55] Vojtech D., Cizkovsky J., Novak P., Serak J., Fabian T. (2008). Effect of niobium on the structure and high-temperature oxidation of TiAl–Ti_5_Si_3_ eutectic alloy. Intermetallics.

[56] Wang H., Zhu C., Zhang J., Cao R. (2018). Influence of Thermal Exposure at Different Temperatures on Room Temperature Tensile Ductility of Cast TiAl Alloy. J. Mater. Eng..

[57] Narayana P.L., Kim J.H., Yun D.W., Kim S., Reddy N.S., Yeom J., Seo D., Hong J. (2022). High temperature isothermal oxidation behavior of electron beam melted multi-phase γ-TiAl alloy. Intermetallics.

[58] Panin P.V., Zavodov A.V., Lukina E.A. (2022). Effect of thermal exposure on microstructure evolution and mechanical properties of cast beta-solidifying TiAl-based alloy doped with Gd. Intermetallics.

[59] Li D., Zhang G., Lu G., Liu Y., Wang J., Liu C. (2022). Precipitation of Ti_2_Al phases at lamellar interfaces in a high-Nb-containing TiAl alloy during thermal exposure. J. Mater. Sci. Technol..

[60] Wang Y., Xue X., Kou H., Qiang F., Yu Y., Yin Z., Li J. (2021). Microstructure Characterization and Thermal Stability of TNM Alloy Fabricated by Powder Hot Isostatic Pressing. Metals.

[61] Zheng G., Tang B., Zhao S., Wang W.Y., Ding Y., Zhao Z., Zhu L., Li J. (2023). Revealing cellular reaction mechanisms and high-temperature structure stability in β-stabilized TiAl alloy. Mater. Charact..

[62] Karadge M., Gouma P.I. (2004). A structural aspect of α(α2) → lamellar α2 + γ transformation in γ-TiAl. Phil. Mag. Lett..

[63] Boehlert C.J., Dimiduk D.M., Hemker K.J. (2002). The phase evolution, mechanical behavior, and microstructural instability of a fully-lamellar Ti–46Al(at.%) alloy. Scr. Mater..

[64] Haase C., Barrales-Mora L.A. (2018). Influence of deformation and annealing twinning on the microstructure and texture evolution of face-centered cubic high-entropy alloys. Acta Mater..

[65] Brice D.A., Rahimi R.M., Bahr D.F. (2019). Hardening Particulate Ti Media Through Controlled Oxidation. Metall. Mater. Trans. A.

[66] Gardner H.M., Gopon P., Magazzeni C.M., Radecka A., Fox K., Rugg D., Wade J., Armstrong D.E.J., Moody M.P., Bagot P.A.J. (2021). Quantifying the effect of oxygen on micro-mechanical properties of a near-alpha titanium alloy. J. Mater. Res..

[67] Göken M., Kempf M., Nix W.D. (2001). Hardness and modulus of the lamellar microstructure in PST-TiAl studied by nanoindentations and AFM. Acta Mater..

[68] Kienl C., León-Cázares F.D., Rae C.M.F. (2022). Deformation twinning during high temperature compression tests of the Ni-base superalloy ATI 718Plus®. Acta Mater..

[69] Amann F., Poulain R., Delannoy S., Couzinié J.P., Clouet E., Guillot I., Prima F. (2023). An improved combination of tensile strength and ductility in titanium alloys via oxygen ordering. Mater. Sci. Eng. A..

[70] Zhu C.L., Li S., Li H.Z., Zhang J. (2014). Effect of Thermal Exposure at 750 °C on Room Temperature Tensile Ductility of Cast Tial Alloy with Directional Lamellar Microstructure. Acta Metall. Sin..

